# Molecular Pathways and Genomic Landscape of Glioblastoma Stem Cells: Opportunities for Targeted Therapy

**DOI:** 10.3390/cancers14153743

**Published:** 2022-07-31

**Authors:** Andrew M. Hersh, Hallie Gaitsch, Safwan Alomari, Daniel Lubelski, Betty M. Tyler

**Affiliations:** 1 Department of Neurosurgery, Johns Hopkins University School of Medicine, Baltimore, MD 21287, USA; ahersh2@jhmi.edu (A.M.H.); hgaitsc1@jhmi.edu (H.G.); salomar1@jhmi.edu (S.A.); dlubelski@jhmi.edu (D.L.); 2 NIH Oxford-Cambridge Scholars Program, Wellcome—MRC Cambridge Stem Cell Institute and Department of Clinical Neurosciences, University of Cambridge, Cambridge CB2 1TN, UK

**Keywords:** glioblastoma, stem cell, targeted therapy, molecular pathway

## Abstract

**Simple Summary:**

Glioblastoma stem cells are a unique population of tumor cells that contribute to tumor growth, invasion, and resistance to chemotherapy and radiation therapy. These stem cells are capable of self-renewal and proliferation. Traditional treatment strategies that target glioblastoma often fail to eradicate these stem cells, contributing to tumor recurrence. Dysregulation of several critical signaling pathways drive the oncogenic nature of glioblastoma stem cells and represent attractive therapeutic targets. Additionally, these stem cells possess a high mutation rate and feature epigenomic changes that alter the landscape of their genomic expression. Here, we review the phenotypic characteristics of glioblastoma stem cells, their interactions with the tumor microenvironment, critical signaling pathways, and epigenomic landscape of glioblastoma stem cells. Therapeutic targets are discussed in the context of these pathways and mutations. A multi-pronged therapeutic approach will likely be needed to simultaneously target multiple pathways and molecules to overcome tumor resistance mechanisms.

**Abstract:**

Glioblastoma (GBM) is an aggressive tumor of the central nervous system categorized by the World Health Organization as a Grade 4 astrocytoma. Despite treatment with surgical resection, adjuvant chemotherapy, and radiation therapy, outcomes remain poor, with a median survival of only 14-16 months. Although tumor regression is often observed initially after treatment, long-term recurrence or progression invariably occurs. Tumor growth, invasion, and recurrence is mediated by a unique population of glioblastoma stem cells (GSCs). Their high mutation rate and dysregulated transcriptional landscape augment their resistance to conventional chemotherapy and radiation therapy, explaining the poor outcomes observed in patients. Consequently, GSCs have emerged as targets of interest in new treatment paradigms. Here, we review the unique properties of GSCs, including their interactions with the hypoxic microenvironment that drives their proliferation. We discuss vital signaling pathways in GSCs that mediate stemness, self-renewal, proliferation, and invasion, including the Notch, epidermal growth factor receptor, phosphatidylinositol 3-kinase/Akt, sonic hedgehog, transforming growth factor beta, Wnt, signal transducer and activator of transcription 3, and inhibitors of differentiation pathways. We also review epigenomic changes in GSCs that influence their transcriptional state, including DNA methylation, histone methylation and acetylation, and miRNA expression. The constituent molecular components of the signaling pathways and epigenomic regulators represent potential sites for targeted therapy, and representative examples of inhibitory molecules and pharmaceuticals are discussed. Continued investigation into the molecular pathways of GSCs and candidate therapeutics is needed to discover new effective treatments for GBM and improve survival.

## 1. Introduction

Glioblastoma (GBM) is the most common and aggressive malignancy of the central nervous system (CNS), accounting for 82% of all malignant gliomas [1]. The annual incidence of GBM is 5.26 per 100,000 population [2]. GBM can occur in both children and adults, although the median age at diagnosis is 65 years and the incidence increases with age [2]. The standard-of-care consists of maximal surgical resection with adjuvant temozolomide (TMZ) chemotherapy and radiation therapy (RT) [3]. Unfortunately, outcomes remain poor, with a median survival of 14-16 months, and the five-year survival rate for GBM patients is less than 5% [3,4]. These outcomes reflect a high recurrence rate greater than 90% and, despite therapeutic intervention, GBM carries an invariably fatal prognosis. The resilience of GBM reflects its infiltrative nature that renders gross total resection difficult, as well as its rapid growth and resistance to therapeutics [3]. 

GBM is classified by the World Health Organization as a Grade 4 astrocytoma [5]. Traditionally, the diagnosis of GBM was based on histological features, including high vascularity, pseudopalisading necrosis, and infiltration into surrounding normal brain tissue. Updated guidelines in 2021 from the World Health Organization have begun to incorporate genetic mutations into the grading system, allowing for GBM to be classified not only based on its histological appearance, but also as an IDH wild-type, H3 wild-type neoplasm with TERT promoter mutation, amplification of the epidermal growth factor receptor gene, or chromosome copy-number alterations. The updated classifications reflect the importance of genetic drivers of tumor growth and progression, and genetic analysis is increasingly being used to stratify tumors, predict prognosis, and individualize therapy [6,7]. 

The majority of GBM tumors are primary and arise de novo, however, some can arise secondarily from lower grade astrocytomas [4]. GBM tumors exhibit high inter- and intra-tumoral heterogeneity at the molecular and cellular levels, due to a broad range of oncogenic driver mutations [8]. GBM tumors can be further classified into classical, mesenchymal, neural, and proneural subtypes based on their transcriptional profiles [9]. 

The rapid mutation rate of GBM often renders treatment with TMZ and RT quickly ineffective, even if initial treatment regimens are successful in achieving tumor regression. In recent decades, cancer stem cells (CSCs) have arisen as potential targets in the development of antineoplastic therapies. CSCs share traits of stem cells found in healthy body tissues, including the ability to self-renew and differentiate into other cell types. CSCs feature in the pathogenesis and progression of cancer, including malignant gliomas [10,11]. Tumor recurrence in GBM is mediated by treatment-resistant glioblastoma stem cells (GSCs) residing in the vicinity of the resection cavity [12,13]. GSCs are preferentially resistant to current chemo- and radiotherapy regimens; therefore, components of their self-renewal and differentiation pathways may be potential targets for future GBM therapeutics [14,15]. This review will provide an overview of GSCs, including their unique features, key signaling pathways, and genomic landscape, and conclude with opportunities for therapeutic targeting. 

## 2. Features of Glioblastoma Stem Cells

GSCs share many attributes with neural stem cells (NSCs), including the ability to self-renew, differentiate, and form neurospheres in culture [15,16]. They also share many of the molecular markers commonly used to isolate and identify NSCs [15]. However, GSCs differ from NSCs in their genetic mutational profiles, chromosomal abnormalities, and tumorigenicity [15,17]. They are also characterized by their low abundance within the tumor and low proliferative activity, which protects them from therapies targeting dividing cells [18]. It remains unclear whether GSCs arise from NSCs or from other CNS cell types with mutational burdens that promote the self-renewal phenotype. Regardless of origin, evidence suggests that GSCs are the driving forces behind GBM recurrence and treatment resistance. The majority of GBM tumors recur within 2 cm of the primary tumor site and these recurring tumors often display a nodular pattern, suggesting derivation from a clonal population [15]. These observations suggest that a subset of lingering primary tumor cells, rather than de novo tumor formation, are responsible for tumor recurrence. 

An in vivo xenograft transplantation study of GSCs demonstrated their high tumorigenicity, with as few as 100 GSCs being sufficient for recapitulating tumor growth [19]. By contrast, transplantation of much larger numbers of differentiated tumor cells were able to engraft but not form tumors [19]. GSCs have also been shown to exhibit radioresistance [20] and chemoresistance [21,22] when compared to non-GSC tumor cells. The resilience of GSCs to these insults is due to aberrant activation of several repair and maintenance pathways [15]. Mutations leading to upregulation of the DNA repair enzyme O6-Methylguanine-DNA methyltransferase (MGMT) have also been shown to trigger chemoresistance [23]. Furthermore, GSCs appear to be resistant to other chemotherapeutics, including carboplatin, paclitaxel, and etoposide [23].

When describing characteristics of GSCs, it is vital to first define the GSC population. The question of which markers to use for GSCs remains controversial. As with traditional stem cell populations, there is first the issue of describing a cellular flux state as cells transition from stem-like progenitors to differentiated progeny. Single-cell RNA-sequencing studies have revealed clusters of cells undergoing small differentiation steps within the overarching GSC population [8]. The search for markers is further complicated by intra-tumoral heterogeneity and newly appreciated tumor subtypes [9,24]. Numerous enrichment markers have been proposed over the past decade, including Nestin [25,26], A2B5 [27], Sox2 [28,29], LGR5 [30], and GPD1 [31]. However, the cell surface glycoprotein CD133 remains the most widely accepted GSC marker [19]. CD133 has high specificity for glioma cells with stem-like characteristics [16,32]. 

Region-specific markers for GSCs have also been explored, including those in the tumor core, tumor rim and peri-tumor (invasive) area. Analysis of resected GBM tissue indicates distinct differences in terms of cell density, cell proliferation, vascularity, and gene expression between each of these areas [33]. CD44, for example, has been identified as a GSC marker in the tumor periphery and has been suggested to play a role in tumor invasion and migration [34]. Knockdown of the surface marker in cells cultured from the tumor edge impairs invasion and migration [34]. Given the limitations of surgical resection and the fact that most recurrence occurs at the periphery of the initial resection cavity, the search for markers at the tumor edge is especially important. The use of multiple markers, including CD133, is desirable and the employment of functional assays to determine “stem-like” glioma cells is crucial. 

In addition to GBM tumor subtyping, GSCs can be categorized as proneural or mesenchymal [35]. The subtype of a primary GBM can change upon tumor recurrence, which may reflect survival of one GSC subpopulation in an initially heterogeneous pool, or conversion from one GSC subtype to another [18,36]. In vitro analysis shows that these two cell populations exhibit differential gene expression of vesicles, resulting in the secretion of extracellular vesicles with vastly different proteomic makeups, which in turn mediate disparate downstream signaling effects [35]. Further exploration of the distinct biological characteristics of GSC subtypes will assist with selection of anti-GSC targets, and multiple therapeutics may be necessary to target heterogeneity within the GSC niche. 

The phenotypic characteristics of GSCs are mediated by a repertoire of signaling pathways and mutations, including loss of function of the tumor suppressor p53. Mutations in the p53 pathway, including somatic missense mutations in the DNA binding domain, are found in over 80% of GBMs [37]. Aside from its involvement overseeing the cell cycle, the p53 tumor suppressor also regulates self-renewal and differentiation of embryonic stem cells, such that loss of function contributes to dedifferentiation of stem cells into GSCs and promotes their stemness and proliferation. Neurospheres deficient in p53 have enhanced self-renewal and proliferative capacities [38]. Moreover, mutant p53 is involved in reprogramming of the GSC metabolic pathway to favor glucose uptake and glycolysis, upregulate expression of oncogenes, and drive an inflammatory response that contributes to further dedifferentiation of cancer cells [39]. Therefore, loss of function of p53 is a critical step in enhancing GSC survival, and consequently, GBM growth.

## 3. Interactions with Tumor Microenvironment

### 3.1. Stem Cell Niches

GSCs engage in reciprocal signaling with their microenvironment, remodeling their milieu while being simultaneously influenced by their surroundings. Four niches characterize the locations of GSCs within GBMs, including perivascular, hypoxic, necrotic, and invasive niches [40]. The tumor microenvironment influences differential expression of GSC phenotypes, resulting in intra-tumoral heterogeneity spatially distributed within the same patient. Tissue samples from patients with GBM have shown differentially expressed values of PD-L1 sampled from the tumor core, periphery, and surrounding brain tissue, with expression in the periphery corresponding to tumor invasion and suppression of the immune system [41]. The constative activation of PD-L1 at the periphery may contribute to therapeutic resistance and tumor progression in patients with subtotal resections, while suggesting that several therapeutics may be needed to target the multiple niches within GBM. 

The perivascular niche features direct contact between GSCs and endothelial cells allowing for supply of oxygen and essential nutrients to GSCs, along with communication between GSCs, reactive astrocytes, pericytes, fibroblasts, and myeloid cells [42]. Signaling between endothelial cells and GSCs promotes stemness, self-renewal, and invasion of the stem cells. Reactive astrocytes produce vascular endothelial growth factor which stimulates neovascularization to supply blood to tumor cells, while GSCs promote vascular dilation and increased leakiness [42]. GSCs, endothelial cells, astrocytes, and microglia also exchange microRNAs that promote angiogenesis in the microenvironment and increase the self-renewal and proliferative capacities of GSCs [40].

The greater proliferation rate of GSCs compared to endothelial cells ultimately results in a necrotic niche consisting of a low population of endothelial cells. A hypoxic niche surrounds the necrotic core and constitutes areas of decreased oxygen delivery. The invasive niche includes areas of tumor infiltration, including migration along vessel walls, rendering gross total resection difficult [42]. Tumor cellular invasion is followed by neovascularization that produces leaky blood vessels and disruption of the BBB by displacement of astrocytes and pericytes. The infiltration of GSCs along the microvasculature is termed “vessel co-option” and incorporates both a vascular and infiltrative niche [43]. Reactive astrocytes further promote tumor invasion through signaling pathways such as the Sonic hedgehog pathway described further below [44]. Indeed, Rath et al. illustrated in vitro that astrocytes promote invasion of GSCs but not CD133- cells by secreting chemokines and cytokines and upregulating GSC genes involved in cellular movement [45].

Intercellular communication between GBM and the tumor microenvironment is achieved via direct exchange through ion channels, transporters, gap junctions, and extracellular vesicles, as well as secretion of cytokines, chemokines, and neurotransmitters [40]. Extracellular vesicles can transport microRNA and proteins that remodel the microenvironment to promote tumor aggressiveness, stimulate angiogenesis, and promote resistance to therapy, including chemotherapeutics and radiation [46]. Moreover, specific GSC subtypes localizing to different niches are shown to produce distinct extracellular vesicles that contribute to intratumoral heterogeneity [47]. Extracellular vesicles released by GSCs also interact with the immune system and contain PD-L1 to suppress T cell activity. Similarly, expression of TrkB on extracellular vesicles released by GSCs promotes therapeutic resistance and tumor progression [48].

### 3.2. Hypoxia Signaling

Hypoxia is a notable characteristic of malignant tumors, which rapidly outgrow their blood supply [49]. Physiological mechanisms in place for responding to a lack of oxygen rely on hypoxia-inducible factors (HIFs), transcription factors that induce expression of a wide range of gene products related to angiogenesis, erythropoiesis, proliferation, and cell survival [49,50]. The two primary HIF isoforms, HIF-1 and HIF-2, are differentially regulated by cellular oxygen levels and have unique target specificities, resulting in a complex network of complementary functions [51,52]. Two HIF subunits, HIF-1α and HIF-2α, are particularly important for tumor maintenance and GSC viability [53,54]. The link between hypoxia signaling and GSC function is further supported by the colocalization of hypoxia-induced and stem cell-associated transcripts in patient GBM tissue [55]. 

The hypoxic tumor microenvironment is a key regulator of GSCs and helps maintain tumor cell stemness and prevent differentiation [51,56,57]. HIF-1α and HIF-2α interact with pathways known to be important in GSC signaling, including the Notch and calcineurin pathways [51,57]. Studies have shown that when HIF-2α-knockdown GBM cell lines are exposed to hypoxic conditions, upregulation of GSC-associated genes fails to occur, indicating that hypoxic cellular changes are mediated by HIF signaling [51,58]. Interestingly, knockdown of HIF-1α does not appear to exert a significant effect on GSC-associated gene expression [51]. However, it does seem necessary for hypoxia-mediated maintenance of GSCs, likely through its activation of the Notch pathway [57,59]. Knockdown of both HIF-1α and HIF-2α in GBM cell lines reduces their sphere-forming ability after exposure to hypoxic conditions, indicating a decrease in self-renewal capability [51]. Analysis of GSCs isolated from human GBM tissue found that increased expression of hypoxia-related genes is associated with a worse clinical prognosis, further indicating the relevance of hypoxia signaling in tumor maintenance and progression [51].

Hypoxia in GBM can be exacerbated by treatment, potentially promoting GSC therapeutic resistance [60]. An in vitro study using patient-derived xenograft recurrent GBM models demonstrated that treatment with TMZ induces HIF-1α and HIF-2α expression that coincides with real-time observation of de-differentiation of non-GSC tumor cells to stem-like cells [58]. This study supports the theory that HIF overexpression following TMZ treatment is partially responsible for treatment resistance by increasing the stemness of the remaining tumor cell population. To further clarify the relationship between hypoxia signaling and chemoresistance, a siRNA approach was used to silence HIF-2α in a TMZ-treated GBM cell line, resulting in a reduction in chemotherapeutic resistance and neurosphere formation [61]. Additionally, GSCs treated with siRNA targeting HIF-2α expressed decreased levels of the stem cell markers Nestin and CD133 [61]. Together, these studies indicate that HIFs may serve as promising targets for anti-GSC therapies [53].

### 3.3. Metabolic Environment

GSCs are characterized by metabolic plasticity and reprogramming as a result of unique tumor mutations and in response to the tumor microenvironment, including the relative supply of oxygen. In addition to promotion of tumor progression, loss of the tumor suppressor gene p53 promotes glycolysis and is found in most GBMs [62]. Similarly, activation of the oncogenic c-MYC protein promotes glycolysis, while loss of function of the isocitrate dehydrogenase genes, key enzymes in the citric acid cycle of oxidative metabolism, are implicated in over 90% of secondary GBM. Some researchers postulate that altered metabolism is in fact a cause, rather than an effect, of oncogenesis [63].

The location of GSCs within particular niches drives their metabolic profiles [64]. Most prominently, GSCs in the hypoxic niche must adapt to the lack of oxygen traditionally used for oxidative phosphorylation. Consequently, GSCs generally rely on non-oxidative glycolysis for their energy needs. HIF-1α signaling plays a prominent role in metabolic pathways by promoting transcription of glucose transporters and the lactate dehydrogenase A protein which diverts pyruvate from the citric acid cycle and instead converts it to lactate [63,65]. Moreover, secretion of pyruvate, lactate, and glutamine from the endothelium in the tumor microenvironment can serve as a source of fuel for hypoxic GSCs [64]. Nonetheless, GSCs are not exclusively reliant on glycolysis, and can indeed generate energy using the citric acid cycle. Indeed, some GSCs contain higher levels of ATP than differentiated GBM cells, complicating the overall picture of metabolic reprogramming and illustrating intratumoral heterogeneity at the metabolic level, while suggesting that purely targeting the glycolytic pathway may not be effective against all GSCs [66,67]. In fact, mutations are found throughout the mitochondrial DNA genome in GBM cells, altering metabolic pathways and energy generation and contributing to therapeutic resistance [68]. Certain GSC subtypes rely on the glycolytic pathway to greater extents than other subtypes, which also utilize the mitochondrial pathways, illustrating the need for multiple therapeutic targets [63,67]. 

In addition, GSCs can activate the anabolic pentose phosphate pathway to produce nucleic acids and fatty acids. The pathway is initially suppressed under hypoxic conditions, resulting in a metabolic switch between glycolysis during periods of hypoxia and the pentose phosphate pathway during periods of oxygenation [69]. Long-term exposure to hypoxia may upregulate pentose phosphate pathway enzymes critical for GSC proliferation [67]. Oxidation of fatty acids can also promote GSC self-renewal and progression [63].

## 4. Signal Transduction Pathways

The contributions of GSCs to tumor pathogenesis are mediated by a diverse repertoire of signaling pathways that influence GSC function and stemness [70]. Molecules in these pathways may serve as the basis of anti-GSC targets. Here, we highlight several important signaling pathways that are known to play a role in GSC survival, proliferation, and tumor recurrence.

### 4.1. Notch

Canonical Notch pathway receptors (Notch 1-4) are cell surface type 1 transmembrane receptors that mediate cell–cell signaling [71]. Notch ligands include delta-like 1, delta-like 3, delta-like 4, Jagged1, and Jagged2 [72]. The binding of ligands triggers the Notch receptors to undergo a series of successive *γ*-secretase-mediated proteolytic cleavage steps, liberating the Notch intracellular domain (NICD) [72]. Binding of the NICD to downstream transcriptional regulators in the nucleus allows Notch signaling to control expression of a broad range of target genes [72]. 

Analogously to other stem cell signaling molecules, Notch plays a key role in early embryogenesis, namely by preventing premature neurogenesis and maintaining pools of progenitor cells in the developing CNS [14]. Notch signaling promotes human brain development by increasing progenitor cell proliferation and astrocyte differentiation [14]. In the adult brain, Notch inhibits NSC apoptosis, promotes self-renewal, and suppresses differentiation, thereby maintaining a functional stem cell reserve in the CNS [14]. Similarly, the Notch pathway is crucial in promoting the survival of GSCs in the tumor microenvironment, and regulates tumor initiation, progression, and recurrence [14,73]. Notch exhibits significant crosstalk with molecules in other signaling pathways, including the PI3K/AKT/mTOR pathway and the hypoxic pathway [74]. It may also exert a role in GSC migration via upregulation of the CXCR4 chemokine [74]. However, the actual sequence of regulatory events and the precise mechanisms through which Notch activity controls stemness and tumorigenicity remain to be elucidated. While the complex role of Notch signaling in the brain remains to be clarified, over-activation of Notch signaling is at least partly responsible for GSC survival.

Computational analysis of largescale patient transcriptional datasets confirms that increased Notch activity correlates with negative clinical outcomes in glioma patients while also serving as a marker for GSC prevalence in tumor tissue [75]. In vitro modulation of Notch signaling further supports the idea that it plays a vital role in GSC vitality. In GSC neurosphere cultures, activation of Notch signaling enhances colony formation, increases self-renewal, and promotes de-differentiation [76]. Additionally, when both Notch and Wnt/β-catenin signaling are inhibited in cultured GSCs, neuronal differentiation is induced and clonogenic potential is inhibited [77]. Thus, targeting proteins involved in the Notch signaling pathway, including NICD, TRIM3, CXCR4, CXCL12, Hes, CPEB1, and Hey—several of which have been implicated in promoting either GSC stemness or differentiation—may be a viable strategy for promoting differentiation of GSCs and thereby sensitizing GBM tumors to therapeutic interventions [14]. However, it is important to note that these effects are only likely to be seen when high endogenous Notch activity can be confirmed in the targeted GSC population or tumor subtype [76].

### 4.2. Epidermal Growth Factor Receptor

Epidermal growth factor receptor (EGFR) is often aberrantly expressed in GBM by amplification or mutation [78]. Increased activity of EGFR, either due to overexpression or the constitutive activity of its deletion variant, EGFRvIII, is associated with more aggressive disease [79]. Like Notch, EGFR plays a role in the maintenance of the NSC population in the CNS [78] and has also been shown to be necessary for the self-renewal capacity of GSCs in culture [80]. Inhibition of EGFR signaling using a tyrosine kinase inhibitor induces GSC differentiation and reduces the tumorigenicity and self-renewal potential of the treated cells [78]. These results indicate that EGFR signaling is necessary for the maintenance of GSCs in an undifferentiated state. Given these findings, anti-EGFR therapies have the potential to target GSCs and may represent a treatment possibility for patients with GBM. However, the GSC population is not homogenous. For instance, coexpression of the variant EGFRvIII, but not EGFR, with CD133 has been reported in GSCs [81]. Thus, any therapeutic interventions focused on signaling pathways must account for GSC heterogeneity.

One downstream signal transduction pathway for EGFR is the phosphatidylinositol 3-kinase (PI3K)/Akt/mammalian target of rapamycin complex (mTOR) survival cascade, which is also genetically altered in the vast majority of GBM tumors [11,82]. While this differential activation frames the PI3K/Akt/mTOR pathway as a promising therapeutic target, in vivo and clinical application of PI3K pathway inhibitors has led to mixed results, possibly due to persistent mTOR signaling [83,84]. Additional studies investigating mTOR signaling escape mechanisms are warranted. Still, in vitro studies of patient derived GSCs indicate that PI3K inhibition reduces stem cell ability to proliferate and invade [85]. Downstream of PI3K, AKT signaling has been identified as a critical factor in the hyperthermia-induced radiosensitization of GSCs, with inhibition of PI3K enhancing these effects [86]. Due to pathway redundancy, it is almost certain that a combination of multiple inhibitors will be required to achieve an anti-GSC effect [87]. Moreover, inhibition of EGFR signaling in GSCs was shown to upregulate related family receptors, including eRBB2 and ERBB3, allowing the GSCs to resist the therapeutic treatment and survive [88]. Therefore, targeting both the EGFR pathway and ERBB family receptors may be necessary to achieve a vigorous anti-tumor response.

### 4.3. Sonic Hedgehog

**Figure 1:**
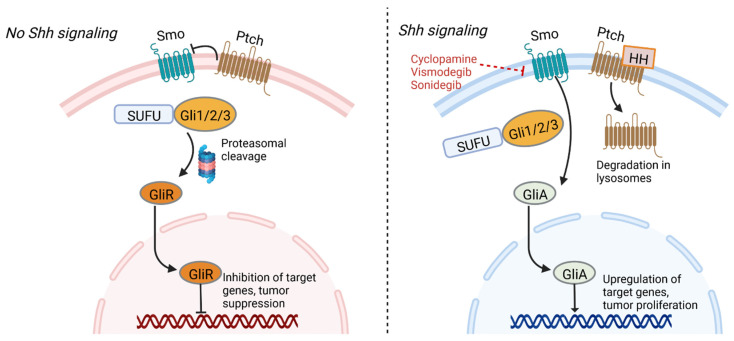
Sonic hedgehog signaling pathway. In the absence of hedgehog ligand, Ptch inhibits Smo, resulting in formation of Gli repressor that inhibits target genes. Ptch and SUFU function as tumor suppressors. Hedgehog ligand’s interaction with Ptch results in its degradation, allowing a signaling cascade mediated by Smo that forms GliA. This transcription factor activates genes associated with tumor proliferation. Inhibitors of Smo represent a potential therapeutic target against GSCs. GliA—Gli activator, GliR—gli repressor, HH—hedgehog ligand, Ptch—Patched, Smo—smoothened, SUFU—Suppressor of fused homolog.

The sonic hedgehog (Shh) signaling pathway plays critical roles in normal embryonic development, neural tube patterning, the maintenance of adult stem cells, and tissue repair [89]. Activation of the pathway involves the sonic hedgehog ligand, the Patched (Ptch) transmembrane receptor, the Smoothened (Smo) transmembrane receptor, and glioma-associated oncogene (Gli) transcription factors. In the absence of hedgehog ligand, Ptch inhibits Smo, resulting in phosphorylation and proteolytic cleavage of the Gli protein into Gli repressor, which in turn binds gene promoters and inhibits their transcription. The binding of hedgehog ligand to the extracellular domain of Ptch results in their internalization and degradation in lysosomes, allowing for Smo to initiate a signaling cascade that allows formation of Gli activator, which then travels to the nucleus and promotes transcription of target genes (Figure 1) [90,91]. 

Shh signaling plays an important regulatory role in cell renewal and cell cycle progression, and signaling components Ptch1, Gli1, and Gli2 are highly expressed in human stem cells and downregulated in differentiated cells [91,92]. Similarly, the pathway is involved in proliferation and self-renewal of cancer stem cells, and genetic expression profiling of GSCs has revealed Shh signaling-dependent pathways in some cell lineages [93,94]. For example, PTEN-expressing GBMs have been shown to contain higher levels of Shh and PTCH1 expression compared to GBMs lacking PTEN expression, and hyperactivity of the signaling pathway and GLI1 over-expression is associated with reduced survival time [91,94]. Inhibition of Shh signaling components could help reduce GBM cell viability. A mouse model using CD133+ GBM cells showed that inhibition of Shh can delay GBM growth and promote apoptosis, while mice overexpressing SHH displayed faster tumor growth [95]. Inhibition of Gli and Smo have also been shown to reduce tumor volume and size of neurospheres grown from GSCs [91,96]. Of note, synergism has been demonstrated using inhibitors against both the sonic hedgehog (SHH) and PI3K/AKT/mTOR pathways, resulting in reduced GSC pluripotency [87].

### 4.4. Transforming Growth Factor Beta

Transforming growth factor beta (TGF-β) is a cytokine involved in embryonic development, control of cell cycle and apoptosis, epithelial to mesenchymal transition, and inflammatory processes [97,98]. As a homeostatic regulator, TGF-β plays a dual role functioning both as a tumor suppressor that promotes cell cycle arrest and apoptosis, as well as an oncogenic factor that promotes cellular invasion and dedifferentiation when the signaling pathway becomes distorted or TGF-β is overexpressed [99,100,101]. TGF-β acts on serine/threonine kinase cell surface receptors, resulting in the assembly of two type I and two type II receptors in an activated heterotetrameric complex [102]. These receptors phosphorylate Smad family proteins, particularly Smad2 and Smad3, which assemble in trimeric complexes with Smad4 for translocation to the nucleus, where they regulate genetic expression [103]. A myriad array of non-Smad signaling pathways can also be activated by TGF-β [99]. 

Smad signaling induces leukemia inhibitory factor which acts via the JAK-STAT pathway to prevent differentiation and promote self-renewal of GSCs but not normal glial stem cells [104]. TGF-β signaling also acts on Sox4 to increase expression of Sox2, a stemness gene responsible for self-renewal. GBM cells around necrotic regions have been shown to express elevated levels of TGF-β along with stem cell markers such as CD133, suggesting that tissue hypoxia may promote TGF-β signaling which induces an epithelial-mesenchymal transition resulting in the stem cell phenotype [105]. In addition, TGF-β prevents proteasomal degradation of Sox9, a protein involved in migration and invasion of GBM cells [106]. Consequently, patients with GBM and high TGF-β/Smad activity tend to have aggressive tumors with a poor prognosis [107]. Inhibitors of TGF-β signaling have illustrated promise as a therapeutic target by promoting GSC differentiation and reducing proliferation [108]. Bone morphogenic proteins, included within the TGF-β family of proteins, also act to reduce the self-renewal capabilities of GSCs and promote their differentiation [14].

### 4.5. Wnt

The Wnt family of glycoproteins are involved in embryonic and neural stem cell development, cellular polarity, cell proliferation, and regulation of stemness [109,110]. In the absence of Wnt ligands, the protein adenomatous polyposis coli (APC) forms a cytoplasmic complex with other proteins that target β-catenin for destruction [111]. The binding of Wnt proteins to the frizzled receptor and low-density lipoprotein receptor-related proteins on the cell surface initiates the signaling pathway, triggering either a canonical or non-canonical signaling cascade. The canonical pathway inhibits the destruction complex, resulting in stabilization of β-catenin, which travels to the nucleus and forms a complex with transcription factors to activate gene transcription. This pathway is involved in stem cell renewal, differentiation, and cellular proliferation [112]. Non-canonical pathways are β-catenin independent and regulate cytoskeletal structure and cell polarity through Rac and Rho GTPases or release of intracellular calcium [110]. Mutations in APC have been widely studied in cancer development, with loss of function of the inhibitory gatekeeper APC seen in nearly 80% of colorectal cancers [113]. Loss of APC also occurs in prostate, breast, gastric, and lung cancer [114]. APC mutations have also been reported in GBM, although their frequency is low, potentially indicating a smaller role for genetic mutations in GBM pathogenesis compared to other tumors [115,116].

Aberrant hyperactivation of the Wnt signaling pathway, whose causes can include mutations in APC, β-catenin, WTX, and TCF4, is implicated in tumor growth, recurrence, and self-renewal of GSCs [117]. The Wnt pathway has also been implicated in GBM resistance to TMZ and RT [118]. FoxM1, a nuclear transcription factor overexpressed in GBM, has been shown to form a complex with β-catenin allowing for accumulation of β-catenin in the nucleus of tumor cells [116]. Overexpression of the oncogene PLAGL2 upregulates Wnt signaling molecules in the canonical pathway and enhances GSC self-renewal by regulating cellular differentiation [119]. In contrast, inhibition of WNT signaling and β-catenin can reduce GSC proliferation and suppress cell migration and invasion [110].

### 4.6. Signal Transducer and Activator of Transcription 3

**Figure 2:**
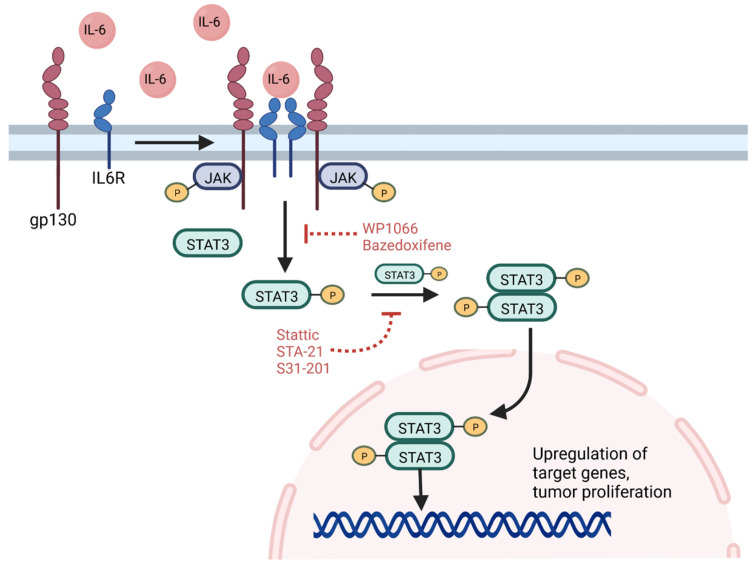
STAT3 signaling pathway. The IL-6 cytokine triggers dimerization and activation of the IL6 receptor with its gp130 subunit. JAK phosphorylation in turn results in STAT3 phosphorylation, which dimerizes and translocates to the nucleus to upregulate target genes associated with stemness and GSC survival. Inhibition of the STAT3 pathway can be achieved at several points, including targeting of receptor signaling using WP1066 or bazedoxifene and inhibition of STAT3 dimerization and signaling using Stattic, STA-21, or S31-201. GP130—glycoprotein 130, IL6R—IL6 receptor, P—phosphorylation, STAT3—signal transducer and activator of transcription 3.

Activation and amplification of the signal transducer and activator of transcription 3 (STAT3) protein has been reported in nearly half of all human tumors and is associated with tumor proliferation, tumor survival, tissue invasion, and immunosuppression [120,121]. STAT3 functions as a signal transducer of cytokine pathways and a transcription factor that regulates expression of hundreds of genes, including oncogenes, and plays additional roles in epigenetic regulation and chromatin remodeling [121]. Canonical STAT3 signaling pathways feature ligand-induced dimerization of cytokine receptor, particularly the IL-6 receptor, which in turn phosphorylates the Janus family kinases (JAKs) [122]. These kinases phosphorylate tyrosine 705 of cytosolic STAT3, resulting in homodimerization and translocation to the nucleus where it serves as a regulator of gene expression (Figure 2) [123]. Other tyrosine kinases, including EGFR, can also phosphorylate STAT3 through similar pathways not initiated by IL-6 [123,124]. Post-translational modifications of STAT3 further influence its nuclear functions [121]. For example, phosphorylation of Enhancer of Zeste Homolog 2 (EZH2) results in STAT3 methylation, increasing STAT3 activity, and this interaction preferentially occurs in GSCs relative to other tumor cells [125]. The suppressor of cytokine signaling 3 protein normally acts to inhibit STAT3 signaling [122]. Recently, non-canonical pathways have been discovered, and some target genes can be activated by unphosphorylated STAT3, which also accumulates in response to IL-6 signaling [126]. 

Constitutive activation of STAT3 has been detected in several cancer stem cells, including prostate, breast, and GBM, where it promotes a stem-cell phenotype and survival [127]. Hypoxic conditions can activate the JAK-STAT signaling pathway, driving GSC self-renewal [128]. A positive feedback loop in GSCs has also been discovered, in which Toll-like receptor 9, a molecule associated with tumor growth, drives activation of STAT3, which in turn upregulates expression of the Toll-like receptor [129]. The receptor is also activated by pathogen-associated molecular patterns, and together with overexpression of the IL-6 receptor in GSCs, may indicate a role for inflammation in oncogenesis and GSC survival [121,127]. The glycoprotein CD109 can also activate the IL-6/STAT3 signaling pathway in GSCs, contributing to GSC self-renewal and tumorigenicity, and depletion of CD109 was shown to impair stemness and contribute to a differentiated phenotype [130]. In addition, RT can promote phosphorylation of STAT3, which can contribute to tumor resistance to radiation. Conversely, inhibition of STAT3 has been shown to promote radiosensitivity in GBM cell lines [123,131].

STAT3 has emerged as a therapeutic target given its significant role in signaling and transcription pathways, although most work has been performed in vitro or in rodent models. Inhibition of STAT3 can affect growth and survival of GBM. Sherry et al. illustrated that inhibition of STAT3 using small molecule inhibitors or genetic knockdown using short hairpin RNA reduces GSC proliferation, neurosphere formation, and markers of GSC multipotency [132]. These results highlight the role of STAT3 in maintaining the phenotype of GSCs. Small molecular inhibitors of STAT3 have also been shown to induce apoptosis of GSCs [133]. 

### 4.7. Inhibitors of Differentiation

Basic helix-loop-helix proteins are transcription factors that form heterodimers and bind promoter and enhancer regions of genes, where they help regulate cell lineage and differentiation [134]. Inhibitors of differentiation (ID) proteins form dimers with basic helix-loop-helix proteins but cannot bind DNA as they lack basic moieties, thereby functioning as negative regulators of these transcription factors and instead serving to maintain the stem cell niche and self-renewal [135,136]. Consequently, ID proteins promote tumorigenesis and are upregulated in GBM, among other cancers, including breast and prostate cancer, where higher levels are associated with poorer prognosis [135,136,137,138,139].

One member of the ID family of proteins, ID-1, is situated at the nexus of multiple signal transduction pathways in GSCs. Cycloxygenase-2, overexpressed in GBM, can induce ID-1 expression via a mitogen-activated protein kinase pathway that upregulates the early growth response protein 1 transcription factor [140]. The ID-1 protein can promote GSC self-renewal by inhibiting Cullin 3, a ubiquitin ligase normally responsible for ubiquitin-mediated degradation of the DVL2 and GLI2 proteins. Suppression of Cullin 3 increases DVL2 and GLI2 levels, which in turn promote noncanonical ligand independent WNT and SHH signaling pathways that drive maintenance of GSCs [139]. Additional research has shown that ID-1-mediated WNT and SHH signaling can increase expression of the *MYC* proto-oncogene, whose functions include promoting transcription of miR-17 and miR-20a. These miRNAs inhibit expression of the bone morphogenetic protein receptor, a member of the TGF-β signaling pathway that promotes differentiation [141]. Downregulation of the receptor promotes resistance to differentiation signals from bone morphogenetic proteins, which normally initiate a signal transduction cascade after binding their type II receptor to promote transcription of target genes [141,142,143]. 

In addition to the Wnt, Shh, and TGF-β signaling pathways, research in other cancers has linked ID-1 to K-ras signaling, PI3K/Akt signaling, and STAT3 signaling, illustrating the centrality of ID-1 across multiple signaling pathways [134]. The influence of ID-1 on these pathways and activation of GSC self-renewal contributes to GSC resistance to chemotherapy and RT, worsening outcomes in patients with high expression of *ID1* [134]. ID-1 can activate EGFR pathways that promote resistance to TMZ chemotherapy, resulting in tumor recurrence with ID-1-enriched cells after treatment [136]. EGFR can also induce a related ID protein, ID-3, which has also been shown to confer a stem cell phenotype to primary astrocytes [144]. Knockout of three genes of the ID family in mice with high-grade gliomas resulted in tumor regression and disruption of the interactions between GSCs and endothelial cells of the perivascular niche [145]. Therefore, therapeutics targeting ID-1 or related ID proteins may represent a potential novel treatment for GBM. 

## 5. Epigenetic Regulation

In addition to changes in signal transduction pathways that influence genetic expression in GSCs, epigenetic regulation plays a vital role in regulating transcription of oncogenes and tumor suppressors. Epigenetic modalities include DNA methylation, histone acetylation, histone methylation, and microRNAs (miRNA), which alter chromatin structure and affect transcription without changing the underlying DNA sequence [146]. DNA methylation is typically associated with genetic silencing and condensed chromatin formation while histone acetylation promotes an open form of chromatin that upregulates genetic transcription [147,148]. Histone methylation is associated with variable effects on transcription depending on the histone location and degree of methylation [149]. Epigenetic perturbations are widespread in cancer and can be used as biomarkers to assess prognosis and treatment response [150]. Epigenetic reprogramming represents a potential therapeutic target in cancer by downregulating the transcription of genes associated with tumor invasion and upregulating genes that are abnormally silenced in cancer cells [151,152].

Epigenetic mechanisms can control expression of genes associated with DNA damage repair, proliferation, stem cell self-renewal, and angiogenesis [153]. A study of 60 patient-derived GSC cultures indicated that stratification into distinct clusters based on epigenetic markers can predict patient survival, highlighting the prognostic influence of epigenetic regulation and heterogeneity across tumor samples [154]. Similarly, identification of methylation markers at CpG sites in GBM samples can predict tumor progression and overall survival [155]. 

### 5.1. DNA Methylation

GSCs possess a unique DNA methylation signature relative to other GBM and neuronal stem cells [156]. Methylated CpG islands have been reported at higher frequencies in GSCs compared to GBM bulk tumors, illustrating the critical and widespread role of DNA methylation in GSCs [157]. Stratification of GSCs based on DNA methylation patterns represents a potential epigenetic biomarker and correlates with clinical prognosis [157]. Recognition of hypermethylated genes in GSCs can suggest candidate tumor suppressors while hypomethylated genes may drive GSC maintenance and tumor invasion. Lee et al. identified 675 genes associated with DNA methylation changes in GSCs, including some that are hypermethylated, such as *SPINT2*, *GATA6*, *NEFM*, and *CCNA1*, and others that are hypomethylated, including *CARD10* and *CLIC3*. In vitro overexpression of one such hypermethylated candidate tumor suppressor, *SPINT2*, reduced tumor sphere formation and proliferation, indicating that evaluating changes in DNA methylation can help identify potential therapeutic targets [156].

### 5.2. Histone Post-translational Modifications

Trimethylation of lysine 9 on histone H3, or H3K9me3, is a repressive marker associated with transcriptional silencing [158,159]. Histone modifiers recruited by H3K9me3 promote formation of heterochromatin [160]. Additionally, H3K9me3 is involved in regulation of cellular differentiation and lineage commitment [161]. For example, H3K9me3 increases during differentiation of oligodendrocytes and in response to differentiating stimuli [162]. Mallm et al. illustrated that inhibition of the histone demethylases KDMC4C and KMD7A via the dimethyloxaloylglycine inhibitor can increase H3K9me3 levels in GSCs, induce differentiation, and repress expression of DNA repair genes, resulting in accumulation of DNA damage that can cause apoptosis [163]. Separately, Liau et al. illustrated that GSCs present in a slow-cycling, persistent state upregulate the histone demethylases KDM6A/B, further suggesting that histone demethylases may represent a therapeutic target in GBM [164].

In contrast, trimethylation of H3K27 is a repressive mark involved in maintenance and self-renewal of stem cells [165]. Trimethylation is mediated by polycomb repressive complex 2, a multimeric histone methyltransferase that includes the EZH2 protein [166,167]. The EZH2 protein catalyzes transfer of a methyl group from S-adenosyl methionine to the lysine side chain of H3 [168]. EZH2 is often overexpressed in cancer, including prostate, breast, and brain cancer, and is a poor prognostic marker in gliomas [169,170]. Consequently, EZH2 inhibitors are of interest as a therapeutic treatment in GBM [168,171,172]. Suvà et al. illustrated that the *MYC* oncogene, essential for GSC maintenance, is a downstream target of EZH2 in GSCs [173]. EZH2 also recruits DNMT1 to hypermethylate the promoter of the bone morphogenetic protein receptor gene, promoting resistance to differentiation signals from bone morphogenetic protein [174]. Additionally, EZH2 can recruit DNA methyltransferases to add methyl groups to DNA, and can also methylate proteins such as STAT3, which promotes the STAT3 signaling pathway in GSCs [175]. 

Histone acetylation neutralizes the charge on histones and induces an open chromatin state that favors genetic transcription [176]. Conversely, histone deacetylases (HDACs) repress transcription by removing acetyl groups from the lysine side chains of histones. Increased levels of *HDAC* expression have been reported in GBM and are associated with shorter survival and higher tumor grades [177,178]. Aberrant expression of *HDAC* genes in GSCs promote stemness and self-renewal [179]. For example, HDAC6 overexpression is associated with activation of the Gli1/Shh signaling pathway, potentially due to Gli1 deacetylation or deacetylation of tubulin required for coordination of Shh signal transduction. Inhibition of HDAC6 in GSCs was reported to downregulate the Shh pathway, induce differentiation, and promote GSC apoptosis [180]. HDACs also increase DNA resistance to damage from therapeutics by maintaining chromatin in a condensed state [181]. Therefore, HDAC inhibitors, such as valproic acid, are an active area of investigation in GBM and may promote GSC differentiation [182].

### 5.3. MicroRNA

MicroRNAs consist of small, single-stranded, non-coding RNA molecules that regulate cellular function, including stemness and tumorigenesis [183]. MiRNAs assemble into the RNA-induced silencing complex, which base pairs with target mRNA to promote their degradation or inhibit translation [183,184]. These non-coding RNA molecules are estimated to regulate nearly 60% of human genes, and dysregulation of miRNA expression features in the initiation and proliferation of cancer [185]. MiRNA can act as tumor suppressors or promoters of oncogenes. Tumor suppressive miRNAs inhibit oncogenic mRNA and are often located in fragile regions of the genome, such that deletions or mutations that silence tumor suppressive miRNAs result in upregulation of oncogenic proteins [185,186]. Conversely, miRNAs that enhance cancer formation often inhibit tumor suppressors, and aberrant expression of these miRNAs can drive tumor growth [187]. MiRNAs are involved in regulation of GSCs and signaling pathways that affect stemness, self-renewal, differentiation, and resistance to therapy [185,187]. Additionally, they regulate translation of transcription factors associated with epigenetic reprogramming that drives tumor growth [187]. 

Several miRNAs feature in GSC regulation. Tomei et al. used array-based profiling and real-time polymerase chain reaction to identify 14 miRNAs differentially expressed in GSCs compared to autologous differentiated tumor cells [188]. These miRNAs regulated the epithelial-mesenchymal transition and signaling pathways, including the Notch signaling involved in maintenance of stemness. Dysregulation of miRNAs, particularly miR-21 and miR-95, were linked to overall survival [188]. Additionally, Sana et al. identified 431 significantly deregulated miRNAs in GSCs compared to differentiated tumor cells, including several linked to markers of stemness [189]. Seven of these miRNAs could predict patient prognosis. Additionally, the hypoxic microenvironment of GSCs amplifies miRNA dysregulation [190,191].

MiRNAs that suppress translation of oncogenic mRNAs are often downregulated in GSCs, while those driving GSC proliferation are upregulated. Downregulation of miR-34a —a noncoding RNA that acts as a tumor suppressor, promotes cellular differentiation, and whose overexpression induces apoptosis — has been reported in GSCs [192]. MiR-34a is also involved in downregulation of the Notch signaling pathway in GSCs and other malignancies [193,194]. Therefore, a decrease in miR-34a promotes GSC maintenance and tumor proliferation. Similarly, miR-181a inhibits the *Notch2* oncogene and reduces the level of the CD133 marker. Downregulation of miR-181a, particularly in association with increased expression of Notch2, is associated with shorter overall survival in GBM [195]. Decreased expression of miR-29a is also reported in GSCs, which normally acts as a tumor suppressor by inhibiting expression of Quaking gene isoform 6 involved in the PI3K/Akt signaling pathway [196]. In contrast, overexpression of miR-29A induces apoptosis and inhibits tumor growth and invasion [197]. Additionally, silencing of miR-137 expression in GSCs helps maintain the stem-cell phenotype and prevents differentiation [198]. miR-124 and miR-137 also induce differentiation and are downregulated in GBM [199]. Maintenance of GSC self-renewal is also regulated by miR-128, which decreases methylation of H3K27 and phosphorylation of Akt, and is decreased in GBM [200]. Furthermore, several miRNAs have been identified that drive tumorigenesis and GSC growth. For example, expression of miR-17 enriches GSCs and increases expression levels of CD133 [201]. 

## 6. Resistance Mechanisms to Therapy

The Stupp protocol outlines the mainstay of treatment for GBM and includes maximal safe surgical resection followed by adjuvant RT and maintenance chemotherapy using TMZ [202,203]. TMZ is an alkylating agent that methylates purine bases. Methylation of guanine at its O^6^ site forms O6-methylguanine and triggers apoptosis of the tumor cell [204]. RT causes cellular damage and the formation of reactive oxygen species, including hydroxyl radicals, that result in DNA double-strand breaks, triggering autophagy, apoptosis, and necrosis [205]. Although the protocol was formulated over 15 years ago, progression and recurrence remain the norm, with a median survival time under 15 months [202]. Therefore, there is significant interest in mechanisms of resistance to TMZ and RT, which may provide insights into novel targets for therapeutic agents. 

Chemotherapy and RT preferentially target differentiated cancer cells and may initially shrink the tumor, but a chemo- and radioresistant GSC population contribute to relapse and progression [206,207,208]. The main resistance mechanisms include repair of DNA damage, induction of anti-apoptotic signaling pathways, efflux of therapeutics, scavenging of reactive oxygen species (ROS), quiescence, and promotion of self-renewal and stemness [209,210]. In addition, heterogeneity across GSCs ensures an array of mutations that improve adaptability and resistance [32]. Here, we detail mechanisms promoting resistance of GSCs to chemotherapy and RT.

### 6.1. Chemoresistance

GSC resistance to chemotherapy contributes to GBM progression after treatment [208]. DNA damage repair pathways are upregulated in GSCs, helping them to evade apoptosis, and include the overexpression of O^6^-methylguanine-DNA methyltransferase, an enzyme that negates the effects of TMZ alkylation by transferring the methyl from the O^6^ site of guanine to its cystine residue [211,212]. Beier et al. illustrated TMZ can effectively target MGMT-negative cells while sparing MGMT-positive GSCs [213]. Inhibition of the pro-apoptotic tumor suppressor p53 from overexpression of the *MDM2* gene also increases resistance to TMZ [212]. Additionally, the DNA mismatch repair pathway responsible for apoptosis after recognizing alkylated base pairs is often dysregulated or inactive in GSCs, limiting the efficacy of TMZ [210]. Genome-wide analysis implicated mutations in four core proteins governing the mismatch repair pathway, including *MLH1*, *MSH2*, *MSH6*, and *PMS2*, in regulating resistance of GSCs to TMZ cytotoxicity [214]. Furthermore, upregulation of DNA repair enzymes, such as poly-ADP-ribose polymerase 1, mitigate damage from TMZ [215,216]. MiRNAs are additionally implicated in GSC chemoresistance. GSCs that upregulate miR-21 and downregulate miR-145 can evade apoptosis in response to chemotherapy [217,218]. Overexpression of miR-455-3p has also been demonstrated in TMZ-resistant GSCs, likely due to suppression of SMAD2 involved in TGF-β signaling by miR-455-3p [219]. Additionally, GSCs express elevated levels of free radical scavenging systems that counteract chemotherapeutics that promote free radical formation and oxidative damage [220,221].

Induction of signaling pathways that counter apoptosis and promote stem cell self-renewal also mitigate the effects of chemotherapy. Upregulation of the Notch, Shh, and Wnt pathways follow TMZ treatment and mediate stem cell maintenance and proliferation [222]. Consequently, inhibition of these pathways sensitizes GSCs to TMZ [222]. Furthermore, TMZ can result in dedifferentiation of cancer cells into GSCs by inducing markers of stemness, enriching the tumor with GSCs, and promoting resistance and invasion [223]. The PI3K/Akt pathway can inhibit pro-apoptotic proteins, such as Bcl-2-associated agonist of cell death protein, while the STAT3 pathway increases *MGMT* expression that removes methyl groups from purines alkylated by TMZ [208]. The hypoxic microenvironment and expression of HIF-1α and HIF-2α also increase *MGMT* expression and augment chemoresistance [220,224,225].

Additional mechanisms influencing GSC chemoresistance include expression of drug efflux transporters. Drug delivery to tumor cells is limited by the tight microvascular network constituting the blood–brain barrier, and those drugs capable of intratumoral accumulation can be removed by ATP-binding cassette transporters [226]. Overexpression of these multi-drug resistance transporters on GSC cell membranes mediate chemoresistance to a range of therapeutics [210,227]. Furthermore, increased levels of major vault protein are reported in GSCs, a constituent of the ribonucleoparticle vault complex that removes drugs from the nucleus and sequesters them in vesicles [228]. Correspondingly, higher levels of the vault protein correlated with poorer prognosis in patients receiving chemotherapy, while knockdown of the protein mitigated chemoresistance [229]. Hypoxic signaling also potentiates the role of transporters in drug efflux [230]. 

### 6.2. Radioresistance

GSCs possess several mechanisms to resist the effects of ionizing radiation, which forms free radicals and damages DNA. The GSC population has been noted to increase after patients undergo radiosurgery for treatment of malignant gliomas, limiting treatment efficacy [231,232]. RT selects for a subpopulation of cancer stem cells with mutations conferring resistance, resulting in clonal expansion of radioresistant stem cells that promote tumor growth and recurrence [232]. Interactions with the tumor microenvironment are critical for enhancing radioresistance, and components of the perivascular niche and extracellular matrix can attenuate the damaging effects of RT [233]. A hypoxic microenvironment, as with chemotherapeutics, promotes resistance to RT, especially as oxygen is needed for stabilization of free radicals and formation of reactive oxygen species that cause DNA damage [232]. Proteins in the extracellular matrix can activate integrin-mediated signaling pathways that increase survival after RT, while tenascin C in the perivascular niche can counteract damage from RT by promoting GSC proliferation [233]. Additionally, differentiated tumor cells can dedifferentiate into GSCs via epithelial-mesenchymal transition in response to RT, suggesting that recurrence and progression of GBM is mediated not only by clonal expansion of native GSCs, but also transformation of differentiated tumor cells [234]. 

Analogously to chemotherapy, RT induces stem-cell signaling pathways that mediate radioresistance. GSCs activate DNA damage repair pathways with greater efficacy than differentiated tumor cells in response to RT [20]. The Wnt pathway can initiate a cascade leading to autophagy, a lysosomal degradation process that allows for adaptation and detoxification in response to stress [235]. Notch signaling and the STAT3 pathway also promote stem-cell resistance, transcription of anti-apoptotic genes, and stem cell maintenance [123,233]. Upregulation of *RAD51*, which plays a crucial role in homologous repair of DNA double-strand breaks, reduces DNA damage after radiation, and its inhibition sensitizes GSCs to RT [227,236]. Moreover, GSCs can differentiate into endothelial cells in response to RT, resulting in angiogenesis and neovascularization that improve survival [227,237]. Additionally, DNA methylation profiling after irradiation of GSC cultures illustrates that extended dose fractionations alter the landscape of DNA methylation. Genes expressing heat shock proteins, histones, and miRNAs are among those differentially methylated targets after radiation. These changes may contribute to GSC survival after radiation exposure [238].

## 7. Targeted Therapy

**Figure 3:**
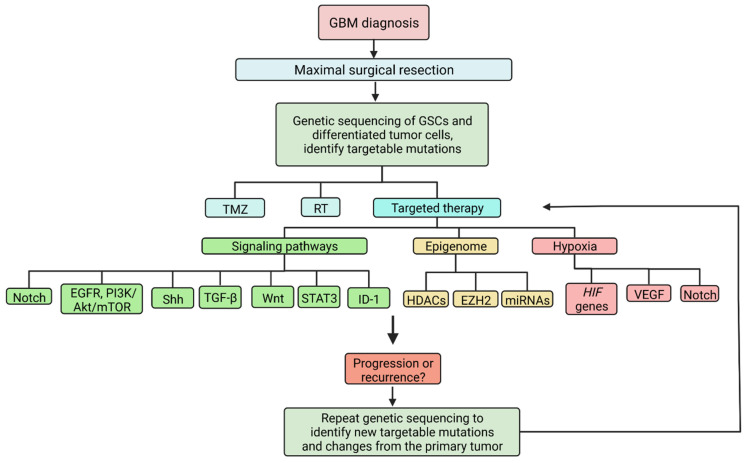
Proposed future management algorithm for GBM. The genomic revolution has offered significant insights into pathogenesis of GBM and resistance to TMZ and RT. Clinicians may eventually have an array of pharmaceutical agents and therapeutics designed to target specific molecular components of tumor cells and GSCs. Following maximal surgical resection, genomic analysis should be performed to identify key signaling pathways and genomic mutations in GSCs that can allow for personalized targeted therapy, performed alongside adjuvant TMZ and RT. Clinicians may eventually select from amongst several drugs those likely to exert the most profound anti-GBM effect based on the genomic analysis. In the case of tumor progression or recurrence, repeat sequencing can be performed to identify new actionable mutations for targeted therapy. Additionally, the targeted therapy may sensitize the tumors to TMZ and RT, and synergistic effects may arise from multiple regimens. Additional therapeutic agents can also be designed to target differentiated tumor cells. EGFR—epidermal growth factor receptor, EZH2—Enhancer of zeste homolog 2, GBM—glioblastoma, HDAC—histone deacetylase, HIF—hypoxia inducible factor, ID-1—Inhibitor of differentiation-1, mTOR—mammalian target of rapamycin, PI3K—Phosphoinositide 3-kinase, RT—radiation therapy, Shh—sonic hedgehog, STAT3—Signal Transducer and Activator Of Transcription 3, TMZ—temozolomide, VEGF—vascular endothelial growth factor.

Identification of the molecular signaling pathways and epigenomic alterations of GSCs can allow for the design of targeted therapeutics directed at the GSC population. Traditional treatment with adjuvant chemotherapy and RT improves outcomes after surgical resection of GBM, but recurrence and progression nearly always occur, largely due to a resistant stem cell population that replenishes the tumor and promotes invasion. The diverse repertoire of key signaling pathways provides several targets for investigation. Genetic analysis after tumor resection may provide insight into the genomic landscape of the GSC population, allowing clinicians to select personalized therapeutics (Figure 3). Here, we summarize efforts at promoting GSC death and improving outcomes in patients with GBM.

### 7.1. Targeting Signaling Pathways

Stem cell self-renewal, proliferation, and survival are mediated by signal transduction cascades whose constituent molecules can be individually targeted to promote cell death. For example, the Notch pathway, which promotes maintenance of the GSC population, can be targeted by *γ*-secretase inhibitors, which prevent the *γ*-secretase-mediated proteolytic cleavage that liberates the NICD after ligand binding to transmembrane receptors. These inhibitors have been shown to reduce neurosphere growth and increase GSC apoptosis [239]. Similarly, the PI3K/Akt signaling pathway can be targeted by metformin and its potent analog phenformin [240,241]. Metformin treatment inhibits Akt signaling and increases AMP-activated protein kinase, which inhibits mTOR, in addition to activating the transcription factor FOXO3, a tumor suppressor that promotes differentiation and is often phosphorylated and silenced by AKT in GSCs [242]. Relatedly, agents such as erlotinib can target the EGFR pathway and the constitutively active EGFRvIII variant to reduce GSC proliferation [243].

The Shh signaling cascade can also be targeted to reduce the GSC population. The alkaloid cyclopamine, responsible for teratogenic birth defects by binding Smo and suppressing Shh signaling in embryogenesis, is being investigated for t2reatment of some solid tumors [93,244]. Other Smo inhibitors are under development. Hung et al. showed that the small molecule SMO antagonist sonidegib (LDE225), a drug approved by the FDA for recurrent basal cell carcinoma, can downregulate the key Shh signaling molecules Ptch1 and Gli1 contributing to autophagy in GSCs [95]. Moreover, they illustrated that GSCs were more sensitive to treatment than CD133^-^ cells, confirming the important contribution of Shh signaling to GSC survival. Vismodegib is another FDA-approved Smo antagonist used in treatment of basal cell carcinoma that can be investigated for applications in GBM [245,246]. 

Inhibition of STAT3, which functions both as a signal transducer and transcription factor, may also improve outcomes in GBM. Small interfering RNAs targeting STAT3 can reduce the GSC population in vitro and tumor growth in vivo [247]. Small molecule inhibitors of the STAT3 pathway include Stattic, which targets the SH2 domain of STAT3, and WP1066, which suppresses STAT3 activity and prevents its phosphorylation [123,248]. Treatment with these inhibitors reduced GSC survival, improved outcomes in mouse models, and sensitized GSCs to RT [123,249]. Indeed, WP1066 has been used in clinical trials. Results from a Phase 1 trial of oral WP1066 for recurrent malignant glioma illustrated a favorable safety profile but failure to prevent disease progression over time, although further studies are ongoing to optimize targeting and combine with additional treatment regimens [250]. Other STAT3 inhibitors under investigation include STA-21 and S31-201, which also target the SH2 domain and prevent DNA binding, and illustrated efficacy depleting the GSC population in vitro [132]. Bazedoxifene, an FDA-approved selective estrogen receptor modulator, improved survival in vivo using orthotopic GBM mouse models [251]. The drug is believed to block downstream signaling from the IL-6 receptor, preventing STAT3 activation in GSCs.

Therapeutic targeting of TGF-β receptors and signaling compounds can induce GSC apoptosis. Xiao et al. demonstrated that statins could reduce TGF-β activity and inhibit Smad signaling [252]. Their in vivo mouse model showed that simvastatin can significantly inhibit the growth of GSCs and prolong survival. The mechanism of action includes inhibition of HMG-CoA reductase by statins, resulting in downstream inhibition of Smad3 phosphorylation by Rho and ROCK [252]. Liu et al. illustrated that the experimental compound galunisertib, which inhibits TGF-β receptor I kinase, can be deployed against GSCs in combination with the anti-alcohol drug disulfiram, which targets aldehyde dehydrogenases and sensitizes GSCs to the TGF-β receptor inhibitor [253]. Another inhibitor of the TGF-β receptor I, LY2109761, was shown to reduce expression of the CD44 stem cell marker in GSCs and reduce tumor growth and recurrence in an in vivo mouse model [254].

Additional signaling pathways of interest include the ID and Wnt pathways. ID1 interacts with several other signaling pathways, including WNT, SHH, TGF-β, PI3K/Akt, and STAT3, rendering it a target of interest in GBM. The aforementioned study of the TGF-β receptor I inhibitor, LY2109761, also illustrated reduced expression of ID1 and ID3, inhibiting tumorigenesis and growth [254]. Several drugs have been studied for targeting constituent components of the ID1 signaling pathway, including tetramethylpyrazine, cannabidiol, and vinblastine, although much of this research has not focused on GBM [134]. Sachdeva et al. illustrated that genetic knockout of *ID1* or treatment with the antipsychotic drug pimozide can decrease EGFR activation, reduce tumor growth, and potentiate the effects of TMZ therapy [136]. Pimozide increases proteasomal degradation of ID-1 by blocking USP1, a ubiquitin-specific protease responsible for deubiqutination of ID-1 [255,256]. Cannabidiol was also illustrated to downregulate *ID-1* expression in an in vivo GBM mouse model and significantly reduced GBM progression and invasion [135]. Small molecule inhibitors of Wnt/β-catenin signaling also reduce GBM growth [257]. Non-steroidal anti-inflammatories are under investigation as Wnt inhibitors for treatment of several solid tumors. Aspirin can phosphorylate β-catenin, resulting in its degradation, and has shown an anti-GBM effect [110,258]. Celecoxib alongside TMZ chemotherapy has also been investigated in recurrent GBM, although the effects on GSCs are unclear [259]. Signaling pathways and targets of inhibition are summarized in Table 1.

### 7.2. Targeting the Epigenome

Aberrant epigenetic regulation contributes to GSC survival and proliferation, and strategies for therapy include HDAC inhibitors, EZH2 inhibition, and targeting of miRNAs. HDAC inhibitors increase acetylation levels, altering transcriptional pathways involved in apoptosis, proliferation, differentiation, and stemness, and upregulating genes silenced by HDACs [179,271,272]. Valproic acid, an FDA approved anticonvulsant and mood stabilizer, can inhibit HDACs [273]. Riva et al. illustrated that short-term treatment with valproic acid can induce differentiation in GSCs followed by growth arrest, although resistance was observed with long-term treatment [182]. A separate HDAC inhibitor, trichostatin A, was shown to be capable of inducing GSC differentiation and reducing proliferation in vitro [271]. Consequently, a review of 236 patients with GBM identified that valproic acid treatment significantly improved overall survival, including compared to patients receiving other anti-epileptics [274]. Similarly, a retrospective analysis of 291 patients confirmed a two-month increase in survival in patients taking valproic acid [275]. Although these clinical studies did not isolate GSCs specifically, the inhibitory effects of valproic acid on GSCs suggests that GSC differentiation and suppression are mediators of the increase in survival. Separately, Asklund et al. illustrated synergistic effects from combining three HDAC inhibitors—valproic acid, vorinostat, and sodium phenylbutyrate—with the proteasome inhibitor bortezomib, suggesting that polypharmacy may be needed to target intra-tumoral mutational heterogeneity and improve survival [276]. Moreover, Singh et al. found that HDAC inhibitors can also contribute to histone methylation, an effect counteracted by the enzyme lysine specific demethylase 1. Inhibition of the demethylase using tranylcypromine combined with HDAC inhibitors synergistically increased GBM cellular apoptosis [277].

The histone methylator EZH2 can also be targeted to remove repression of silenced genes, including tumor suppressors. GSK343, a selective EZH2 inhibitor, successfully inhibited H3K27 methylation in an in vivo mouse model and reduced tumor growth [278]. Similarly, Jin et al. illustrated that EZH2 inhibitors are particularly effective against proneural GSCs, suggesting that a polypharmaceutical approach may be warranted here as well that targets multiple GSC subtypes [279].

A variety of miRNAs are dysregulated in GSCs and contribute to stemness, maintenance, and proliferation. Identification of upregulated miRNAs in GSCs can inform design of inhibitory therapeutics, while downregulated miRNAs can be exogenously introduced as tumor suppressors. Wong et al. illustrated that miR-31 and miR-148a promote maintenance of GSCs and activate angiogenesis via the HIF-1 pathway [280]. Consequently, their inhibition in a xenograft mouse model reduced tumor growth and improved survival. Esposito et al. used aptamers to deliver miR-137, a tumor suppressor, and anti-miR-10b, which targets the oncogenic miR-10b [281]. They showed that the two compounds combine to reduce tumor growth in vitro. Other studies have reported that inhibition of miR-17-92 and overexpression or treatment with miR-124 and miR-137 can promote cellular differentiation and apoptosis [199,210,282].

### 7.3. Targeting the Tumor Microenvironment

Bidirectional signaling between the tumor and microenvironment is crtiical for GSC proliferation and progression, representing another target for therapeutics. One strategy under investigation targets the hypoxic pathway, which regulates genetic expression and epigenetic modifications to support GSC self-renewal, promote tumor growth and angiogenesis, and increase therapeutic resistance [49,63]. Downregulation of HIF-related genes, such as HIF-1α and HFI-2α, and targeting the hypoxic pathway’s interactions with Notch signaling may sensitize tumors to adjuvant therapy and reduce angiogenesis [283]. Notch signaling is critical for maintaining stemness in hypoxic conditions; [54] therefore, *γ*-secretase inhibitors targeting the Notch pathway may help counteract the effects of hypoxia [239]. 

Inhibitors of vascular endothelial growth factor, such as the FDA-approved monoclonal antibody bevacizumab, are being studied in GBM to target the perivascular niche and counteract the proliferation driven by neovascularization [284]. Indeed, bevacizumab has been used clinically since 2009 as a salvage therapy in recurrent GBM, conferring a four-month improvement in survival [285,286]. Studies have also shown a cytotoxic effect against GSCs, as would be expected from its anti-angiogenic effects [287]. However, treatment resistance is associated with an increase in stem cell markers and a mesenchymal phenotype, along with upregulation of genes promoting tumor invasion [288,289]. Moreover, no benefit has been clearly shown for bevacizumab treatment of primary GBM, and the short improvement in survival for recurrent GBMs illustrates a critical need for continued investigation [290].

Other strategies target the altered metabolic pathways of GSCs. Inhibitors of mutant variants of the *IDH* gene, which produce the oncometabolite D-2-hydroxyglutarate from α-ketoglutarate, can promote differentiation and reduce tumor growth in secondary GBM that have a high occurrence rate of *IDH* mutations [291]. However, targeting mutations in *IDH* is not a viable strategy for primary GBM. Metformin can inhibit the oxidative phosphorylation pathway in mitochondria, targeting the subset of GSCs reliant on oxidative metabolism [63]. Conversely, blockade of the glycolytic pathway can be particularly effective against GSCs and differentiated tumor cells reliant on the Warburg effect. Indinavir and ritonavir are antagonists of the GLUT1 transporter that can reduce GSC proliferation in vitro, although strategies are needed to overcome the blood–brain barrier and localize therapy [67]. Pelaz et al. designed a peptide that targets c-Src, an oncoprotein that interacts with gap junction proteins to regulate gluocse metabolism and promote GSC survival and invasion. The peptide successfully impaired metabolic plasticity and decreased GSC survival [292]. Other metabolic pathways critical for GSC survival besides glucose metabolism can also be targeted. Wang et al. showed that GSCs upregulate the pyrimidine biosynthesis pathway and that targeting critical enzymes in the pathway can impair GSC survival [293].

### 7.4. Challenges

Despite the identification of numerous molecular targets for GBM and promising results in laboratory investigations, treatment outcomes remain poor, and the standard treatment regimen still consists of surgical resection with adjuvant TMZ and RT. Heterogeneity within the GSC population and a high mutation rate often render therapeutic agents effective only for short durations, conferring small increases in overall survival but failing to prevent long-term progression. Polypharmacy may be warranted to simultaneously inhibit multiple signaling pathways, reshape the epigenome, and inhibit angiogenesis. Improvements in genetic sequencing of tumor samples and liquid biopsies may allow personalized therapy, whereby clinicians select from an array of approved drugs those expected to confer the maximum benefit and target the patient’s mutations. Additionally, although in vitro and in vivo studies in rodents may illustrate promising effects using genetic knockdown or introduction of exogeneous miRNA, translating these findings into a pharmaceutical compound can be more elusive. Therapeutics must be rigorously investigated for toxicity and systemic effects. For example, several HDAC inhibitors are known to cause thrombocytopenia, anemia, and hepatotoxicity, and the side effects of novel agents must be balanced with their anticipated effect on patient survival [294]. Furthermore, most investigations into GSCs have focused on intracranial GBM. Although spinal cord GBM is associated with similarly poor overall survival, the generalizability of intracranial results to intramedullary pathology is unclear, and investigations into spinal cord tumors are warranted [295].

Additionally, the blood–brain barrier poses a formidable challenge to the delivery of anti-tumor therapeutics [226]. Uptake of drugs is limited by tight junctions and adherens junctions, as well as multidrug efflux transporters along the barrier. Moreover, therapeutic agents capable of crossing the barrier should be targeted specifically to tumor cells to limit side effects. Nanoparticle encapsulation may represent one tool to improve drug delivery and can be loaded with surface ligands targeting tumor cells. A study of liposomal nanoparticles carrying the antineoplastic agent paclitaxel showed that GSCs could be effectively targeted to reduce proliferation in vivo [296]. Bozzato et al. identified several nanosystems in development for GSC therapy, including polymeric nanoparticle harboring miRNA or siRNA [297]. Continued investigation into drug formulation, toxicity, tumor uptake, and efficacy are needed to translate laboratory findings into clinical practice.

## 8. Conclusions

Despite surgical resection, TMZ chemotherapy, and RT, overall survival in patients with GBM is poor. Tumor progression, invasion, and resistance to therapeutics is mediated by a population of GSCs that harbor a unique genomic and epigenomic landscape in comparison to differentiated tumor cells. These genomic and epigenomic changes drive upregulation of oncogenes and downregulation of tumor suppressors while promoting resistance to conventional therapeutics. Critical interactions with the GSC microenvironment and perivascular niche further support GSC maintenance and proliferation, including the hypoxic pathway, which induces expression of HIF-related genes and interacts with the Notch signaling pathway. Each of these molecular pathways represent potential targets for novel therapeutics, and laboratory investigations have shown promising results with inhibition of key constituents of the signaling pathways. Additional opportunities for targeted therapy include restoration of normal epigenetic regulation and inhibition of the hypoxic pathway. Therapeutic regimens directed against GSCs may eventually be combined with agents targeting differentiated tumor cells to improve overall and progression-free survival in patients with GBM.

## Figures and Tables

**Table 1:** Key signaling pathways identified in GSCs and representative inhibitors.

Signaling Pathway	Inhibitor	Mechanism of Action	Effects of Inhibition	Clinical Trials	References
Notch	*γ*-secretase inhibitors (eg RO4929097, DAPT)	Inhibits proteolytic release of the Notch intracellular domain	Reduces neurosphere growth and GSC markers, prevents tumor growth and improves survival in vivo	NCT01122901, NCT01189240, NCT01269411	[239]
EGFR, PI3K/Akt/mTOR	Metformin	Induces metabolic stress → activates AMPK → inhibits mTOR, activates tumor suppressor FOX03 silenced by signaling pathway	Induces GSC differentiation, inhibits tumor formation and proliferation, improves survival	NCT03243851, NCT02780024, NCT04945148	[219]
	Phenformin		Inhibits GSC self-renewal, increases miRNA expression, inhibits tumor growth and improves survival in vivo		[241]
	Sorafenib	Inhibits receptor tyrosine kinases and PI3K/Akt signaling molecules	Reduces stemness markers, induces apoptosis in GSCs; however, use alongside TMZ did not improve efficacy	NCT00544817, NCT00445588, NCT00884416	[260,261]
	Erlotinib	EGFRvIII inhibitor	Reduces GSC proliferation, synergistic effects with cyclopamine	NCT00301418, NCT01110876, NCT00039494, NCT00274833, NCT00387894	[243,262]
Shh	Cyclopamine	Smo inhibitor	Reduced tumor growth in vitro, depleted GSC population, pretreatment blocked GBM growth in vivo		[93,244,263]
	Vismodegib	Smo inhibitor	Anti-tumor agent for solid tumors and medulloblastoma; treatment with arsenic trioxide and TMZ reduced GBM growth in vivo	NCT00980343, NCT03158389	[264]
	Sonidegib (LDE225)	Smo inhibitor	Downregulated Ptch1 and Gli1, delayed GBM growth in vivo, CD133+ cells most sensitive	NCT01576666—included GBM and several advanced solid tumors	[95]
TGF-β	Statins (eg simvastatin, atorvastatin)	Smad3 inhibition	Inhibited GSC growth and prolonged survival in vivo	NCT02029573—examined atorvastatin	[252]
	Galunisertib (LY2157299)	TGF-β receptor I inhibitor	Disulfiram sensitizes resistant GBM to galunisertib; Phase I study showed clinical benefit in 12/56 patients	NCT01220271	[253,265]
	LY2109761		Decreases CD44 marker and tumor growth		[254,266]
Wnt	Non-steroidal anti-inflammatories	Phosphorylates β-catenin → degradation	Diclofenac and celecoxib reduce GBM proliferation in vitro, downregulate β-catenin activity,	NCT00047281, NCT00112502	[259,267]
STAT3	WP1066	STAT3 inhibitor	Potentiated effects of radiation, improves survival in vivo	NCT01904123	[123,249,250]
	Bazedoxifene	IL-6 receptor inhibitor	Decreases GSC self-renewal capacity, improves survival in vivo		[251]
	Stattic	SH2 domain of STAT3 inhibitor	Sensitizes tumor to radiation and TMZ		[123,130]
	STA-21		Inhibit STAT3 binding to DNA, prevent neurosphere formation, decrease proliferation		[132]
	S31-201				
	STX-0119	STAT3 dimerization inhibitor	Inhibits expression of STAT3 target genes, induces apoptosis, inhibits GSC growth		[268]
ID-1	Cannabidiol	Downregulates ID-1	Reduces GBM invasiveness and self-renewal in vivo, sensitizes GBM to TMZ	NCT01812616, NCT03607643, NCT03529448	[135,269]
	Pimozide	Impairs ID-1 deubiquitination → ID-1 degradation	Sensitizes GBM to TMZ and RT, prolongs time to recurrence in vivo		[136,270]
	LY2109761	TGF-β receptor I inhibitor → reduces ID1 and ID3	Inhibits GBM growth		[254,266]

## Abbreviations

**APC**	Adenomatous polyposis coli
**CNS**	Central nervous system
**CSC**	Cancer stem cell
**EGFR**	Epidermal growth factor receptor
**EZH2**	Enhancer of Zeste Homolog 2
**FDA**	Food and Drug Administration
**GBM**	Glioblastoma multiforme
**GSC**	Glioblastoma stem-like cell
**HDAC**	Histone deacetylase
**HIF**	Hypoxia inducible factor
**JAK**	Janus family kinase
**MGMT**	O6-Methylguanine-DNA methyltransferase
**MiRNA**	MicroRNA
**MTOR**	Mammalian target of rapamycin
**NICD**	Notch intracellular domain
**NSC**	Neural stem cell
**PI3K**	Phosphatidylinositol 3-kinase
**ROS**	Reactive oxygen species
**RT**	Radiation therapy
**SHH**	Sonic hedgehog
**STAT3**	Signal transducer and activator of transcription 3
**TGF-β**	Transforming growth factor beta
**TMZ**	Temozolomide
